# Prevalence and Effects of Functional Vitamin K Insufficiency: The PREVEND Study

**DOI:** 10.3390/nu9121334

**Published:** 2017-12-08

**Authors:** Ineke J. Riphagen, Charlotte A. Keyzer, Nadja E. A. Drummen, Martin H. de Borst, Joline W. J. Beulens, Ron T. Gansevoort, Johanna M. Geleijnse, Frits A. J. Muskiet, Gerjan Navis, Sipke T. Visser, Cees Vermeer, Ido P. Kema, Stephan J. L. Bakker

**Affiliations:** 1 Top Institute Food and Nutrition, 6709 PA Wageningen, The Netherlands; marianne.geleijnse@wur.nl (J.M.G.); s.j.l.bakker@umcg.nl (S.J.L.B.); 2 Department of Internal Medicine, Division of Nephrology, University of Groningen, University Medical Center Groningen, 9700 RB Groningen, The Netherlands; c.a.keyzer@umcg.nl (C.A.K.); m.h.de.borst@umcg.nl (M.H.d.B.); r.t.gansevoort@umcg.nl (R.T.G.); g.j.navis@umcg.nl (G.N.); 3 Department of Laboratory Medicine, University of Groningen, University Medical Center Groningen, 9700 RB Groningen, The Netherlands; f.a.j.muskiet@umcg.nl (F.A.J.M.); i.p.kema@umcg.nl (I.P.K.); 4 R&D Group, VitaK, Maastricht University, 6229 EV Maastricht, The Netherlands; nadja.drummen@vitak.com (N.E.A.D.); cees.vermeer@outlook.com (C.V.); 5 Department of Epidemiology and Biostatistics, EMGO+ Institute for Health and Care Research, VU Medical Center, 1081 BT Amsterdam, The Netherlands; j.beulens@vumc.nl; 6 Julius Center for Health Sciences and Primary Care, University Medical Center Utrecht, 3508 AB Utrecht, The Netherlands; 7 Division of Human Nutrition, Wageningen University, 6700 AA Wageningen, The Netherlands; 8 Unit of PharmacoEpidemiology & PharmacoEconomics, Department of Pharmacy, University of Groningen, 9713 AV Groningen, The Netherlands; sipkev@gmail.com

**Keywords:** vitamin K, matrix Gla protein, all-cause mortality, cardiovascular mortality

## Abstract

Matrix Gla Protein (MGP) is a strong vitamin K-dependent inhibitor of soft tissue calcification. We assessed the prevalence of functional vitamin K insufficiency, as derived from plasma desphospho-uncarboxylated MGP (dp-ucMGP), and investigated whether plasma dp-ucMGP is associated with all-cause and cardiovascular mortality in a large general population-based cohort. We included 4275 subjects (aged 53 ± 12 years, 46.0% male) participating in the prospective general population-based Prevention of Renal and Vascular End-Stage Disease (PREVEND) study. The prevalence of functional vitamin K insufficiency (i.e., dp-ucMGP > 500 pmol/L) was 31% in the total study population. This prevalence was significantly higher among elderly and subjects with comorbidities like hypertension, type 2 diabetes, chronic kidney disease, and cardiovascular disease (~50%). After 10 years of follow-up, 279 subjects had died, with 74 deaths attributable to cardiovascular causes. We found significant J-shaped associations of plasma dp-ucMGP with all-cause (linear term: hazard ratio (HR) (95% confidence interval (CI)) = 0.20 (0.12–0.33), *p* < 0.001; squared term: 1.14 (1.10–1.17), *p* < 0.001) and cardiovascular mortality (linear term: 0.12 (0.05–0.27), *p* < 0.001; squared term: 1.17 (1.11–1.23), *p* < 0.001). These associations remained significant after adjustment for potential confounders. Whether the correction of vitamin K insufficiency improves health outcomes needs to be addressed in future prospective intervention studies.

## 1. Introduction

Along with aging of the population and the rising prevalence of lifestyle-related diseases like type 2 diabetes and hypertension, the prevalence of cardiovascular disease has steadily risen all over the world [1]. Vascular calcification is common in patients with diabetes, hypertension, and chronic kidney disease (CKD), and results in a substantially increased risk for cardiovascular disease (CVD) and mortality [2].

Active matrix Gla protein (MGP) is a strong endogenous inhibitor of soft tissue calcification [3]. Vitamin K is an essential co-factor for the γ-carboxylation of matrix Gla protein (MGP), turning inactive uncarboxylated MGP into active MGP [4]. Vitamin K occurs as two forms in the diet: vitamin K_1_ (phylloquinone) and vitamin K_2_ (menaquinones). Vitamin K_1_ is mainly found in green leafy vegetables and vitamin K_2_ is mainly found in fermented foods such as cheese and natto [5]. Vitamin K insufficiency results in increased plasma levels of inactive uncarboxylated MGP proteins [6]. Plasma desphospho-uncarboxylated (dp-ucMGP) was found to be particularly sensitive for changes in vascular vitamin K status [7].

Furthermore, high plasma dp-ucMGP levels, indicative of functional vitamin K insufficiency, were found to be associated with an increased risk for CVD [8] and mortality [9,10,11,12] in patients with diabetes [8], CKD [9,10], and CVD [11,12]. A recent study demonstrated that high plasma dp-ucMGP concentrations were associated with an increased risk for mortality in the general population [13]. However, data regarding the prevalence of functional vitamin K insufficiency, and thus its clinical impact, are incomplete.

Our objectives were to assess the prevalence of vitamin K insufficiency, as derived from dp-ucMGP, in a large general population-based cohort and to investigate whether vitamin K status is associated with all-cause and cardiovascular mortality. In addition, we aimed to specify a cut-off value for dp-ucMGP to identify subjects at risk for all-cause and cardiovascular mortality.

## 2. Materials and Methods

### 2.1. Study Design and Population

The PREVEND (Prevention of Renal and Vascular End-Stage Disease) study is a prospective cohort study designed to investigate the association of microalbuminuria with renal and cardiovascular disease in the general population. Details of the PREVEND study have been described elsewhere [14,15]. In brief, from 1997 to 1998, all inhabitants of the city of Groningen, the Netherlands, aged 28–75 years (*n* = 85,421) were sent a questionnaire and a vial to collect a first-morning void urine sample. Pregnant women and subjects with type 1 diabetes were excluded. The urinary albumin concentration was assessed in 40,856 responders. Subjects with a urinary albumin concentration ≥10 mg/L (*n* = 7768) were invited to participate in the cohort, of whom 6000 were enrolled. In addition, a randomly selected group with a urinary albumin concentration of <10 mg/L (*n* = 3394) was invited to participate in the cohort, of whom 2592 were enrolled. These 8592 individuals form the PREVEND cohort. The PREVEND study has been approved by the medical ethics committee of the University Medical Center Groningen. All participants provided written informed consent.

The second examination round of the PREVEND study took place from 2001 to 2003, which included 6894 subjects and was considered the ‘baseline’ for the present study. For the present study, we included subjects with available dp-ucMGP measurements and available data on the use of vitamin K antagonists at baseline, leaving 4275 subjects for analyses.

### 2.2. Data Collection and Measurements

The procedures at each examination in the PREVEND study have been described in detail previously [15]. In brief, each examination included two visits to an outpatient clinic separated by 3 weeks. Before the first visit, all participants completed a self-administered questionnaire regarding demographics, cardiovascular and renal disease history, smoking habits, and medication use. Information on medication use was combined with information from IADB.nl [16], a database comprising pharmacy-dispensing data from all community pharmacies in the city of Groningen, the Netherlands, since 1999. During each examination and during each visit, blood pressure was measured on the right arm, every minute for 10 and 8 min, by an automatic Dinamap XL Model 9300 series device (Johnson-Johnson Medical, Tampa, FL, USA). The mean of the last two recordings from each of the two visits was used. Fasting blood samples were provided and stored at −80 °C. 

Vitamin K status was assessed by measuring dp-ucMGP in ethylenediaminetetraacetic acid (EDTA) plasma using a dual-antibody enzyme-linked immunoassay (InaKtif MGP (IDS-iSYS) assay). Serum creatinine (SCr) was measured using an enzymatic method on a Roche Modular analyzer (Roche Diagnostics, Mannheim, Germany). Serum cystatin C concentrations were determined by Gentian Cystatin C Immunoassay (Gentian AS, Moss, Norway) on a Roche Diagnostics Modular auto-analyzer. Cystatin C was calibrated using the standard supplied by the manufacturer (traceable to the International Federation of Clinical Chemistry Working Group for Standardization of Serum Cystatin C). The combined creatinine- and cystatin C-based Chronic Kidney Disease Epidemiology Collaboration (CKD-EPI) equation was used to obtain the estimated glomerular filtration rate (eGFR) [17]. Urinary albumin concentration was measured by immunonephelometry (Dade Behring Diagnostic, Marburg, Germany).

Functional vitamin K insufficiency was defined as a dp-ucMGP concentration >500 pmol/L [7]. Type 2 diabetes was defined as a fasting serum glucose level >7.0 mmol/L, a non-fasting plasma glucose level >11.1 mmol/L, self-report of a physician diagnosis, or the use of glucose lowering drugs, retrieved from a central pharmacy registry. Hypertension was defined as systolic blood pressure ≥140 mmHg or diastolic blood pressure ≥90 mmHg according to the 7th Joint National Committee (JNC 7) [18]. Chronic kidney disease (CKD) was defined as an eGFR <60 mL/min/1.73 m^2^ and/or a urinary albumin excretion (UAE) ≥30 mg/24 h.

### 2.3. Clinical End Points

In the present study, we examined the association of vitamin K status, as derived from dp-ucMGP, with all-cause and cardiovascular mortality. Data on mortality were obtained from the municipal register, and the cause of death was obtained by linking the number of the death certificates to the primary cause of death as coded by a physician from the Central Bureau of Statistics. Cause of death was coded according to the 10th Revision of the International Classification of Diseases (ICD-10). Survival time was defined from baseline until the date of last examination that participants attended, death, relocation to an unknown destination, or 1 January 2011 (end of follow-up).

### 2.4. Statistical Analyses

Statistical analyses were performed using SPSS version 22.0 for Windows (IBM Corporation, Chicago, IL, USA), STATA version 13.1 (StataCorp LP, College Station, TX, USA), and R version 3.0.1 (Vienna, Austria) (http://cran.r-project.org/). Results were expressed as means ± standard deviation (SD) or median (interquartile range (IQR)) for normally and non-normally distributed data, respectively. Nominal data were presented as the total number of patients (percentage). A two-sided *p* < 0.05 was considered to indicate statistical significance.

Baseline characteristics are presented for the total study population and as tertiles of baseline dp-ucMGP concentrations for illustrative purposes and to identify variables (i.e., potential confounders) that are associated with plasma dp-ucMGP in cross-sectional analyses. *p*-values for differences between tertiles were assessed using ANOVA for normally distributed data, Kruskal-Wallis test for skewed data, and the χ^2^ test for nominal data. The χ^2^ test was used to compare the prevalence of a low vitamin K status among subjects with and without comorbidities.

We used Cox regression analyses to investigate the prospective association of baseline dp-ucMGP concentrations with mortality. We applied log_2_ transformation of dp-ucMGP values so the hazard ratios derived from Cox regression analyses were expressed as an increase in risk per doubling of baseline dp-ucMGP values. Cox regression analyses with restricted cubic splines (RCS) with 3 knots were used to depict the J-shaped association of dp-ucMGP with mortality. Cox regression analyses and the STATA package mfpigen were used to test for potential interactions between continuous variables.

Several subjects had missing values for one or more baseline variables (i.e., race, smoking, education, body mass index (BMI), systolic and diastolic blood pressure, UAE (≤1%), total cholesterol/high-density lipoprotein (HDL) cholesterol ratio (3.3%), eGFR (5.4%), and high sensitive C-reactive protein (hs-CRP) (6.0%)). Because excluding subjects with missing values could result in bias, multiple imputation (fully conditional specification (MCMC)) was used to obtain five imputed datasets [19,20]. Rubin’s rules were used to obtain pooled estimates of the regression coefficients and their standard errors across the imputed datasets [21].

Various Cox regression models were built to adjust for possible confounders. The first model depicts the univariable association of log_2_ dp-ucMGP with outcomes; model 2 was adjusted for age and sex; model 3 was additionally adjusted for race, smoking, education, BMI, systolic blood pressure, cholesterol-HDL ratio, ln hs-CRP, type 2 diabetes, use of antihypertensive drugs, and use of vitamin K antagonists; model 4 was additionally adjusted for eGFR and ln UAE. As sensitivity analyses, we additionally adjusted for the presence or absence of a UAE >10 mg/L to test for a potential sampling effect. In further sensitivity analyses, we repeated the Cox regression analyses in subjects that did not use vitamin K antagonists at baseline.

Furthermore, we aimed to identify a cut-off value for dp-ucMGP to identify subjects at increased risk for mortality based on the quantitative measurement of dp-ucMGP. To identify the optimal cut-off, we used the method described by Liu et al. [22], which is available in the STATA package cutpt. This method is a nonparametric approach to identify the optimal cut point for a diagnostic test, which is based on the area under the receiver operating characteristic (ROC) curve, and maximizes the product of the sensitivity and specificity [22].

## 3. Results

### 3.1. Baseline Characteristics

**Figure 1:**
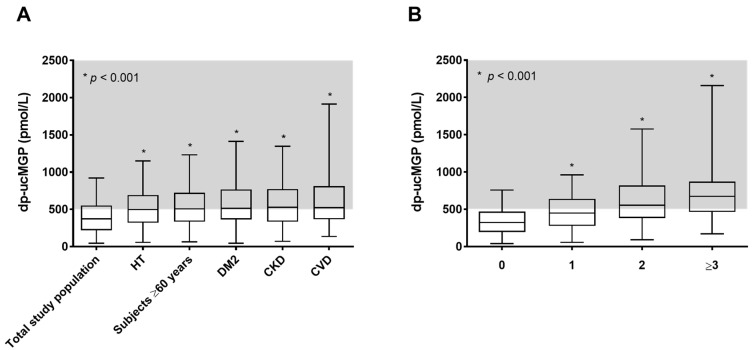
Dp-ucMGP levels in the total study population and for subjects with hypertension (HT), ≥60 years of age, type 2 diabetes (DM2), chronic kidney disease (CKD) and history of cardiovascular disease (CVD) (**A**); and dp-ucMGP levels according to the number of comorbidities (i.e., HT, DM2, CKD, and/or CVD) (**B**).

In the present study, we included 4275 subjects (aged 53 ± 12 years, 46.0% male). Out of all included subjects, 927 (21.7%) had hypertension, 1306 (30.5%) were ≥60 years of age, 84 (2.0%) had type 2 diabetes, 712 (16.7%) had CKD, and 308 (7.2%) had a history of CVD. Baseline characteristics of the total study population and according to tertiles of baseline dp-ucMGP concentrations are presented in Table 1. Median dp-ucMGP concentrations for the total study population (372 (221–552) pmol/L), for subjects ≥60 years old (506 (336–724) pmol/L), and for subjects with hypertension (496 (319–693) pmol/L), type 2 diabetes (514 (364–766) pmol/L), CKD (529 (335–773) pmol/L) and CVD (522 (366–812) pmol/L) are depicted in Figure 1. Dp-ucMGP concentrations were significantly higher in elderly and subjects with comorbidities such as hypertension, type 2 diabetes, CKD, or CVD, and increased even further as the number of comorbidities increased (Figure 1). The prevalence of functional vitamin K insufficiency (i.e., dp-ucMGP > 500 pmol/L) was 31% in the total study population. This prevalence was significantly higher among the elderly and subjects with comorbidities like hypertension, type 2 diabetes, chronic kidney disease, and cardiovascular disease (~50%).

### 3.2. Dp-ucMGP and Mortality

After a median follow-up time of 8.5 (IQR 8.0–9.1) years, 279 subjects (6.5%) had died, with 74 (1.7%) deaths attributable to cardiovascular causes. We found significant deviances from linear associations of dp-ucMGP with all-cause and cardiovascular mortality (both *p*-values < 0.001). In line with the best fitting fractional polynomial model, we included a linear and quadratic term in the Cox regression models predicting mortality.

**Figure 2:**
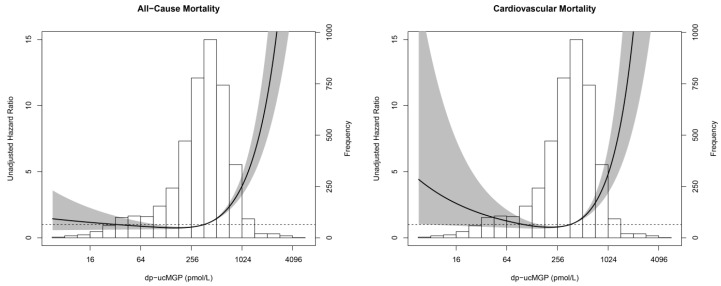
Restricted cubic spline depicting the J-shaped association of dp-ucMGP with all-cause and cardiovascular mortality. The line in the graph represents the risk for all-cause and cardiovascular mortality. The grey area represents the 95% CI of the HR.

The J-shaped associations of dp-ucMGP with all-cause and cardiovascular mortality are shown in Figure 2 and Table 2. Plasma dp-ucMGP was significantly associated with all-cause and cardiovascular mortality. These associations remained significant after adjustment for potential confounders (Table 2). The associations of dp-ucMGP with mortality remained materially unchanged after further adjustment for the presence or absence of a UAE > 10 mg/L. Furthermore, the associations of dp-ucMGP with mortality remained materially unchanged after exclusion of subjects that used vitamin K antagonists (Supplementary Table S1).

Finally, we specified a cut-off value for dp-ucMGP to identify subjects at risk for mortality. The optimal cut-off value was 414 pmol/L for all-cause mortality and 557 pmol/L for cardiovascular mortality.

## 4. Discussion

In the present study, we assessed the prevalence and clinical impact of functional vitamin K insufficiency in a large general population-based cohort. We demonstrated that the prevalence of functional vitamin K insufficiency, as derived from plasma dp-ucMGP, was ~30% in the total study population. Among the elderly and subjects with hypertension, type 2 diabetes, CKD, and CVD, this prevalence was significantly higher (i.e., ~50%), and this prevalence increased even further as the number of comorbidities increased. Furthermore, we found J-shaped associations of plasma dp-ucMGP concentrations with all-cause and cardiovascular mortality. These associations remained significant after adjustment for potential confounders.

Previous studies demonstrated that plasma dp-ucMGP concentrations are significantly higher in healthy subjects aged 66–80 years compared with subjects aged 25–40 years [7], and that plasma dp-ucMGP concentrations are markedly increased in patients with cardiovascular and renal disease [6,9,10,12]. In line with these findings, we found that plasma dp-ucMGP concentrations were significantly higher among elderly and subjects with comorbidities like hypertension, type 2 diabetes, CKD, and CVD. Furthermore, we demonstrated that these dp-ucMGP concentrations increased even further as the number of comorbidities increased.

High plasma dp-ucMGP levels may reflect a low dietary intake of vitamin K, which may contribute to functional vitamin K insufficiency. Currently, the recommended intake of vitamin K is 90 µg/day for women and 120 µg/day for men [23]. However, Booth et al. [24] showed that mean vitamin K_1_ intakes of 1112 men and 1479 women participating in the Framingham Heart Study were 153 ± 115 µg/day and 171 ± 103 µg/day, respectively, implicating that vitamin K intake was inadequate in approximately 40% of the male participants and 20% of the female participants. Cheung et al. [25] showed that vitamin K intake was inadequate in 72% of participants with CKD from NHANES III. Furthermore, Fusaro et al. [26] demonstrated in a controlled observational study that vitamin K_1_ intake was lower in hemodialysis patients compared with controls. Thus, inadequate intake may, at least partly, explain the high prevalence of functional vitamin K insufficiency.

Furthermore, current recommendations on intake of vitamin K are exclusively based on vitamin K_1_, which is the major dietary form of vitamin K in most Western countries, and its function in coagulation [27]. However, these recommended intake values do not reflect vitamin K_2_ and may be suboptimal for the extra-hepatic functions of vitamin K [27,28]. In addition, several studies have suggested that vitamin K_2_, the major dietary form of vitamin K in Japan, may be more effective in activating extra-hepatic vitamin K-dependent proteins than vitamin K_1_ [27]. 

Other important factors that may contribute to functional vitamin K insufficiency are the composition of the intestinal flora [29] and impaired vitamin K recycling [30]. Kaesler et al. [30] demonstrated in a preclinical study that the activity of γ-glutamyl carboxylase, the enzyme that catalyzes the posttranslational γ-carboxylation of vitamin K-dependent proteins such as MGP, was reduced in CKD. This resulted in increased levels of serum undercarboxylated MGP, indicative of functional vitamin K insufficiency [30]. Recent findings of McCabe et al. [31] also illustrated that vitamin K metabolism is altered in CKD, and that this is evident early in the disease course. Furthermore, Kaesler et al. [30] demonstrated that vitamin K supplementation restored intrarenal γ-glutamyl carboxylase activity and reduced heart and kidney calcification. Thus, impaired vitamin K recycling may contribute to functional vitamin K insufficiency in CKD. However, it is not known whether vitamin K recycling is also affected in subjects with, for example, CVD.

If vitamin K status is insufficient, γ-carboxylation of vitamin K-dependent proteins like osteocalcin (OC) and MGP will be impaired, resulting in increased levels of inactive undercarboxylated OC and MGP, which subsequently results in impaired bone metabolism and enhanced vascular calcification [28]. Furthermore, high plasma dp-ucMGP concentrations were found to be associated with an increased mortality risk in patients with aortic stenosis [11], CVD [12], CKD [9], end-stage renal disease (ESRD) [10], and renal transplant recipients (RTR) [6]. Recently, Liu et al. found that plasma dp-ucMGP levels were log-linearly associated with cardiovascular mortality and curvilinearly associated with all-cause mortality in a general population cohort [13]. In line with these findings, we found that plasma dp-ucMGP levels were curvilinearly associated with all-cause and cardiovascular mortality in this large Dutch general population-based cohort.

Furthermore, we aimed to specify a cut-off value for dp-ucMGP to identify subjects at risk for mortality using a nonparametric approach based on the area under the ROC curve described by Liu et al. [22]. We demonstrated that dp-ucMGP levels above 414 pmol/L were associated with an increased risk for all-cause mortality, and those above 557 pmol/L were associated with an increased risk for cardiovascular mortality. These findings are in good agreement with the recent findings of Liu et al. [13], demonstrating that plasma dp-ucMGP levels above 437 pmol/L were associated with an increased risk for all-cause mortality. Although the cut-off value for vitamin K insufficiency of 500 pmol/L may seem arbitrary, the risk of all-cause and cardiovascular mortality strongly increases above this threshold, suggesting that this cut-off value is indeed clinically relevant.

Some limitations of the present study need to be addressed. First, given the observational nature of this study, it is impossible to draw a definite conclusion about the causality of the association of dp-ucMGP with mortality. However, a recent Mendelian randomization study [13] suggests that the association of dp-ucMGP with coronary events and non-cancer mortality is causal. This study, however, might have been underpowered in the Mendelian randomization analysis to detect a significant causal association of dp-ucMGP with cardiovascular death [13]. Furthermore, data regarding vitamin K intake or plasma vitamin K concentrations were not available in this study population, and therefore we could not distinguish between the two forms of vitamin K. However, plasma dp-ucMGP was found to be a sensitive marker for changes in vascular vitamin K status [7] and is considered to be a functional marker of bioactivity of both vitamin K_1_ and K_2_ [5]. Major strengths of the present study are the prospective design, the large sample size, and the long-term follow-up.

## 5. Conclusions

This study provides insights into the prevalence of functional vitamin K insufficiency and its clinical implications in a large general population-based cohort. We demonstrated that functional vitamin K insufficiency is common in the general population and occurs even more frequently among the elderly and subjects with hypertension, type 2 diabetes, CKD, and CVD. Furthermore, we found that plasma dp-ucMGP was curvilinearly associated with an increased risk for all-cause and cardiovascular mortality. Importantly, a low vitamin K status is not only a clinically relevant risk factor for adverse health outcomes, but may also be a modifiable risk factor. Given the availability of vitamin K supplements, vitamin K insufficiency seems an attractive target for preventive intervention. Future prospective clinical trials are needed to investigate whether the correction of low vitamin K status can indeed improve health outcomes.

## Figures and Tables

**Table 1:** Baseline characteristics of the study population.

	All Subjects (*n* = 4275)	Tertiles of dp-ucMGP			*p*-Value
		Tertile 1 (*n* = 1425)	Tertile 2 (*n* = 1425)	Tertile 3 (*n* = 1425)	
dp-ucMGP (pmol/L)	372 (221–552)	<275	275–479	≥480	-
**Demographics**					
Male gender (*n*, %)	1966 (46.0)	570 (40.0)	669 (46.9)	727 (51.0)	<0.001
Age (years)	53 ± 12	49 ± 11	52 ± 11	59 ± 12	<0.001
Race					0.03
Caucasian (*n*, %)	4041 (94.5)	1333 (93.5)	1343 (94.2)	1365 (95.8)	
Black (*n*, %)	42 (1.0)	21 (1.5)	13 (0.9)	8 (0.6)	
Asian (*n*, %)	100 (2.3)	36 (2.5)	36 (2.5)	28 (2.0)	
Other (*n*, %)	59 (1.4)	27 (1.9)	21 (1.5)	11 (0.8)	
Education					<0.001
High (*n*, %)	1431 (33.5)	566 (39.7)	504 (35.4)	361 (25.3)	
Middle (*n*, %)	1015 (23.7)	366 (25.7)	340 (23.9)	309 (21.7)	
Low (*n*, %)	1814 (42.4)	489 (34.3)	576 (40.4)	749 (52.6)	
Smoking (*n*, %)	1206 (28.2)	472 (33.1)	448 (31.4)	286 (20.1)	<0.001
Type 2 diabetes (*n*, %)	84 (2.0)	16 (1.1)	19 (1.3)	49 (3.4)	<0.001
History of CVD (*n*, %)	308 (7.2)	47 (3.3)	86 (6.0)	175 (12.3)	<0.001
**Clinical measurements**					
BMI (kg/m^2^)	26.7 ± 4.3	25.5 ± 3.9	26.4 ± 4.0	28.1 ± 4.5	<0.001
SBP (mmHg)	126 ± 19	121 ± 17	124 ± 18	133 ± 21	<0.001
DBP (mmHg)	73 ± 9	71 ± 9	73 ± 9	75 ± 9	<0.001
**Laboratory parameters**					
Total cholesterol (mmol/L)	5.4 ± 1.1	5.3 ± 1.0	5.5 ± 1.1	5.5 ± 1.1	<0.001
HDL cholesterol (mmol/L)	1.3 ± 0.3	1.3 ± 0.3	1.3 ± 0.3	1.2 ± 0.3	<0.001
Total cholesterol-HDL ratio	4.5 ± 1.3	4.3 ± 1.3	4.5 ± 1.3	4.7 ± 1.2	<0.001
Triglycerides (mmol/L)	1.1 (0.8–1.6)	1.0 (0.8–1.5)	1.1 (0.8–1.5)	1.2 (0.9–1.7)	<0.001
hs-CRP (mg/L)	1.4 (0.6–3.1)	1.1 (0.5–2.9)	1.2 (0.6–2.7)	1.8 (0.9–3.6)	<0.001
UAE (mg/day)	8.1 (5.9–13.4)	7.6 (5.7–11.4)	7.8 (5.8–12.0)	9.3 (6.3–17.9)	<0.001
Serum creatinine (µmol/L)	85 ± 22	81 ± 13	83 ± 14	90 ± 31	<0.001
eGFR (mL/min/1.73 m^2^)	85 ± 16	90 ± 14	87 ± 14	76 ± 17	<0.001
**Medication**					
Vitamin K antagonists (*n*, %)	106 (2.5)	5 (0.4)	6 (0.4)	95 (6.7)	<0.001
Antihypertensive drugs (*n*, %)	990 (23.2)	228 (16.0)	252 (17.7)	510 (35.8)	<0.001
Lipid-lowering drugs (*n*, %)	459 (10.7)	98 (6.9)	130 (9.1)	231 (16.2)	<0.001

Abbreviations: BMI, body mass index; CVD, cardiovascular diseases; dp-ucMGP, desphospho-uncarboxylated matrix Gla protein; DBP, diastolic blood pressure; eGFR, estimated glomerular filtration rate; HDL, high-density lipoprotein; hs-CRP, high sensitive C-reactive protein; SBP, systolic blood pressure; UAE, urinary albumin excretion.

**Table 2:** Associations of log_2_ dp-ucMGP with all-cause and cardiovascular mortality.

	All-Cause Mortality (*n*_events_/*n*_total_ = 279/4275)		Cardiovascular Mortality (*n*_events_/*n*_total_ = 74/4275)	
	HR (95% CI)	*p*-Value	HR (95% CI)	*p*-Value
**Model 1**				
Linear term	0.20 (0.12–0.33)	<0.001	0.12 (0.05–0.27)	<0.001
Squared term	1.14 (1.10–1.17)	<0.001	1.17 (1.11–1.23)	<0.001
**Model 2**				
Linear term	0.27 (0.15–0.47)	<0.001	0.14 (0.06–0.38)	<0.001
Squared term	1.10 (1.06–1.13)	<0.001	1.13 (1.07–1.20)	<0.001
**Model 3**				
Linear term	0.36 (0.18–0.72)	0.004	0.15 (0.04–0.48)	0.002
Squared term	1.07 (1.03–1.12)	0.002	1.13 (1.04–1.22)	0.003
**Model 4**				
Linear term	0.33 (0.17–0.66)	0.002	0.17 (0.05–0.58)	0.004
Squared term	1.08 (1.03–1.13)	0.001	1.11 (1.03–1.20)	0.009

Model 1: crude. Model 2: adjusted for age and sex. Model 3: as model 2 + race, smoking, education level, BMI, SBP, cholesterol-HDL ratio, ln hs-CRP, type 2 diabetes, history of cardiovascular disease, use of antihypertensive drugs, and use of VKA. Model 4: as model 3 + eGFR and ln UAE. Abbreviations: CI, confidence interval; eGFR, estimated glomerular filtration rate; HDL, high-density lipoprotein; HR, hazard ratio; hs-CRP, high sensitive C-reactive protein; SBP, systolic blood pressure; UAE, urinary albumin excretion; VKA, vitamin K antagonists.

## Supplementary Materials

The following are available online at www.mdpi.com/2072-6643/9/12/1334/s1, Table S1: Associations of log_2_ dp-ucMGP with all-cause and cardiovascular mortality after the exclusion of subjects who used vitamin K antagonists.
