# Supplementary material for: Prevalence and Effects of Functional Vitamin K Insufficiency: The PREVEND Study
Source: Nutrients. 2017 Dec 8;9(12):1334. doi: 10.3390/nu9121334 (PMC5748784; doi:10.3390/nu9121334)
Supplement: Supplementary file 1 [file nutrients-09-01334-s001.pdf]

Supplementary Table S1. Associations of $\log_2$ dp-ucMGP with all-cause and cardiovascular mortality after exclusion of subjects who used vitamin K antagonists.

	**All-Cause Mortality (n_{events}/n_{total} = 222/4,169)**		**Cardiovascular Mortality (n_{events}/n_{total} = 57/4,169)**	
	**HR (95% CI)**	***P*-value**	**HR (95% CI)**	***P*-value**
**Model 1**				
Linear term	0.24 (0.12-0.47)	<0.001	0.11 (0.04-0.28)	<0.001
Squared term	1.12 (1.07-1.17)	<0.001	1.18 (1.11-1.25)	<0.001
**Model 2**				
Linear term	0.38 (0.16-0.88)	0.02	0.10 (0.03-0.36)	0.001
Squared term	1.07 (1.01-1.13)	0.02	1.16 (1.07-1.27)	0.001
**Model 3**				
Linear term	0.32 (0.14-0.73)	0.007	0.08 (0.02-0.31)	<0.001
Squared term	1.08 (1.02-1.14)	0.006	1.17 (1.07-1.29)	0.001
**Model 4**				
Linear term	0.30 (0.13-0.69)	0.005	0.10 (0.02-0.40)	0.001
Squared term	1.08 (1.03-1.14)	0.003	1.15 (1.05-1.27)	0.003

Model 1: crude.
Model 2: adjusted for age and sex.
Model 3: as model 2 + race, smoking, education level, BMI, systolic blood pressure, cholesterol-HDL ratio, type 2 diabetes, history of cardiovascular disease, and use of antihypertensive drugs.
Model 4: as model 3 + eGFR and ln UAE.

Abbreviations: eGFR, estimated glomerular filtration rate; HDL, high-density lipoprotein; UAE, urinary albumin excretion.
